# Patient Empowerment During the COVID-19 Pandemic by Ensuring Safe and Fast Communication of Test Results: Implementation and Performance of a Tracking System

**DOI:** 10.2196/27348

**Published:** 2021-06-07

**Authors:** Gunnar Völkel, Axel Fürstberger, Julian D Schwab, Silke D Werle, Nensi Ikonomi, Thomas Gscheidmeier, Johann M Kraus, Alexander Groß, Martin Holderried, Julien Balig, Franz Jobst, Peter Kuhn, Klaus A Kuhn, Oliver Kohlbacher, Udo X Kaisers, Thomas Seufferlein, Hans A Kestler

**Affiliations:** 1 Institute of Medical Systems Biology Ulm University Ulm Germany; 2 Department of Clinical Chemistry University Hospital Ulm Ulm Germany; 3 Department of Medical Development and Quality Management University Hospital Tübingen Tübingen Germany; 4 University Hospital Ulm Ulm Germany; 5 Comprehensive Cancer Center University Hospital Ulm Ulm Germany; 6 Institute of Medical Informatics, Statistics and Epidemiology Technical University of Munich Ulm Germany; 7 Institute for Translational Bioinformatics University Hospital Tübingen Tübingen Germany; 8 Department of Internal Medicine I University Hospital Ulm Ulm Germany

**Keywords:** process optimization, patient empowerment, data security, COVID-19, clinical information system, platform independent, eHealth, telemedicine, quality management

## Abstract

**Background:**

Overcoming the COVID-19 crisis requires new ideas and strategies for online communication of personal medical information and patient empowerment. Rapid testing of a large number of subjects is essential for monitoring and delaying the spread of SARS-CoV-2 in order to mitigate the pandemic’s consequences. People who do not know that they are infected may not stay in quarantine and, thus, risk infecting others. Unfortunately, the massive number of COVID-19 tests performed is challenging for both laboratories and the units that conduct throat swabs and communicate the results.

**Objective:**

The goal of this study was to reduce the communication burden for health care professionals. We developed a secure and easy-to-use tracking system to report COVID-19 test results online that is simple to understand for the tested subjects as soon as these results become available. Instead of personal calls, the system updates the status and the results of the tests automatically. This aims to reduce the delay when informing testees about their results and, consequently, to slow down the virus spread.

**Methods:**

The application in this study draws on an existing tracking tool. With this open-source and browser-based online tracking system, we aim to minimize the time required to inform the tested person and the testing units (eg, hospitals or the public health care system). The system can be integrated into the clinical workflow with very modest effort and avoids excessive load to telephone hotlines.

**Results:**

The test statuses and results are published on a secured webpage, enabling regular status checks by patients; status checks are performed without the use of smartphones, which has some importance, as smartphone usage diminishes with age. Stress tests and statistics show the performance of our software. CTest is currently running at two university hospitals in Germany—University Hospital Ulm and University Hospital Tübingen—with thousands of tests being performed each week. Results show a mean number of 10 (SD 2.8) views per testee.

**Conclusions:**

CTest runs independently of existing infrastructures, aims at straightforward integration, and aims for the safe transmission of information. The system is easy to use for testees. QR (Quick Response) code links allow for quick access to the test results. The mean number of views per entry indicates a reduced amount of time for both health care professionals and testees. The system is quite generic and can be extended and adapted to other communication tasks.

## Introduction

After the first outbreak of SARS-CoV-2 in December 2019 in Wuhan, Hubei province, China, the virus spread rapidly worldwide [1,2]. Thus, the management of its induced crisis has become a ubiquitous topic [3]. Each day, the number of new infections increases worldwide, and reached 107,021,165 confirmed cases globally on February 10, 2021, at 4:22 PM [4].

The current pandemic has significantly affected the possibility of direct interpersonal communication, together with a massive overload of the public health care system. In this context, digital technologies have become crucial sources of support. While surveys and data collection have empowered the evaluation of the first lockdown measures [5], the development of apps and dashboards is paramount in controlling the virus’s spread [6]. Here, rapid case identification is one of the demanding tasks in controlling its spread. The most common available test for COVID-19 infections is to take a throat swab and test by real-time reverse transcription–polymerase chain reaction (RT-PCR) [7-9]. While speeding up this diagnostic test itself has limited feasibility, the time lag between administration of the test and the communication of results can be improved to ensure adequate isolation of positive cases. For ensuring maximal containment of spread, different digital solutions have been applied: (1) rapid identification of cases has been supported by apps concerning contact tracing and tracking of self-reported symptoms [10-14] and (2) special attention has been paid to artificial intelligence approaches supporting home-based self-testing and diagnosis [10,14]. In the case of Germany, available official apps to support health management have been developed and are provided in Table 1 [15-25]. Nevertheless, given the lack of standardization of home-based testing and self-made diagnosis [26,27], empowering the communication between testees and public health institutions is crucial in overcoming the current pandemic crisis. Here, the natural delay between hospital-based tests and the resulting communication with testees has not yet been addressed. Timely communication of results is essential for taking appropriate action; however, it is challenging, due to the high throughput and the high demand under the circumstances in which the tests are carried out. In most European countries, the current practice is to call the respective clinic or laboratory for information about test results [28]. This process ties up considerable resources and does not scale well for large numbers of tests [28]. Communication channels become overloaded and time-consuming for medical staff due to the testees’ repeated calls, as waiting times and anxiety of the testees increases, altogether affecting patient empowerment.

**Table 1:** Official COVID-19–associated apps released in Germany.

App	Institution of release in Germany	Function
Corona-Warn-App [15]	Federal Government, Robert Koch Institute	Anonymized contact tracking Incidence and case reporting Personalized contact tracking
Corona-Datenspende [16]	Robert Koch Institute	Collection of provided health data from fitness trackers and smartwatches
CovApp [17]	Charité and Data4Life	Anonymized questionnaire providing contacts and guidance
Warn-App NINA [18]	Federal Department of Civil Protection and Disaster Assistance	Basic information about the COVID-19 crisis Local valid regulations
CoCoV app [19]	University Hospital Ulm	Private diary on the tolerability of COVID-19 vaccination based on a standardized questionnaire Aggregated analysis and presentation of events that have occurred on a public dashboard
Corona Check [20]	University Hospital Würzburg	Self-screening of symptoms with recommendations for action based on the results Live ticker with official regulations Recommendations on how to protect oneself and others
Corona Health app [21]	University Hospital Würzburg	Anonymized questionnaires on the psychological and physical health-related impacts of COVID-19 Screening analysis News
Safe Vac 2.0 [22]	Paul-Ehrlich-Institut	Anonymized tracking of tolerability of COVID-19 vaccination
WHO Academy: Covid-19 Learning [23]	World Health Organization	Guidance for case management, infection prevention control, and laboratory testing Epidemiology Regional and international information and regulations Research and development
Gutenberg COVID-19 Studien app [24]	University Medical Center Mainz	Survey of study participants
CoronaBoXX [25]	Charité	Health documentation of people in quarantine Hygiene checklist

**Figure 1:**
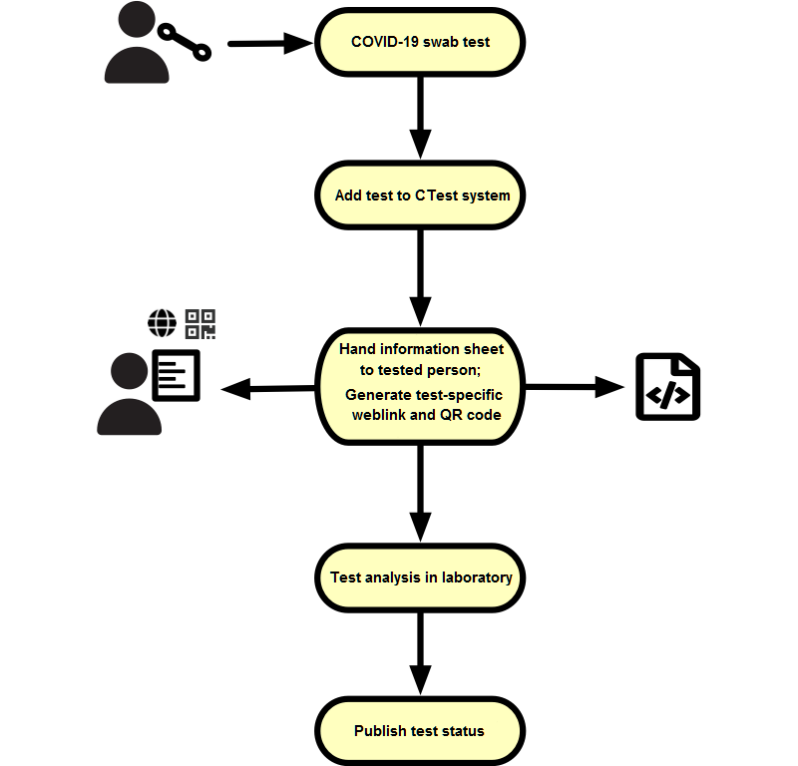
Workflow of the COVID-19 test process using CTest. First, a COVID-19 swab test is performed. The test is then added to the CTest system via an order number. Based on this order number, CTest generates a cryptographically secure tracking ID. An information sheet with a test-specific weblink and QR (Quick Response) code is generated by CTest and handed over to the person tested. After laboratory analysis is complete, the results are sent to CTest. CTest updates the test results, which can be queried via the individual weblink.

We set up a framework to ensure fast and secure communication of health-related results to testees. This was applied to accelerate the COVID-19 testing procedure. Therefore, we implemented an online query system, called CTest, that provides convenient digital access to the status and results of the respective COVID-19 tests to testees (Figure 1). This approach avoids unnecessary and repeated phone calls, avoids manual transcription errors, limits language-associated barriers, and, consequently, reduces the burden on the clinical staff. CTest empowers testees, since they can check their test results and statuses independently through a secure online system [29].

The CTest system extends the functionalities of the previously developed online tracking tool TraqBio [30]. We created this application to simplify and standardize communication between users and core facilities. Its clean design and its open-source license allow for the development (ie, refactoring), deployment, establishment, and integration of CTest within a short period.

CTest runs on Java Virtual Machine [30,31]. The web application can be deployed independently of the operating system and platform.

## Methods

### Implementation and Setup

We based the CTest application on the TraqBio software [30]. The back-end functionality of the CTest server was implemented in the Lisp dialect Clojure. Clojure runs on Java Virtual Machine (Oracle) [31] and is, thus, platform independent. The back end comprises a database that stores the scheduled COVID-19 tests and corresponding results. Clojure supports different databases and connectors. We chose to use SQLite, version 3.29.0 [32], to keep the setup independent of additional database servers. A local database file holds the data. Additionally, CTest provides functionality for user management and data backup.

Every new order number entered and supplied by the laboratory staff in the CTest system generates a database entry with a unique identifier and unique tracking number. Here, this order number is used as the primary unambiguous identifier. CTest can be configured to append the current date to the order number to create a primary unambiguous identifier in setups where order numbers are only guaranteed to be unique within the same day. Using a secure random number generator, we generate a corresponding tracking number for each new entry [33]. This random number generator generates 6 bytes, which are then translated into a sequence of 12 characters containing numbers from 0 to 9 and letters from A to F. This sequence of characters is unique and is not created in sequential order. Every tracking number generated is checked for uniqueness prior to use (ie, there is no link between the tracking number and the order number).

Web-based front-end functionality is implemented using JavaScript and HTML templates. We used freely available standard web frameworks, such as Bootstrap v3 (Twitter) [34,35], jQuery (OpenJS Foundation) [36], and extensions to these frameworks. Using these state-of-the-art frameworks, we aim to enable a straightforward adaptation of the front end for integration at other institutions. The front end features a responsive graphical user interface for the management functions, the creation of tests, and accessibility for users to their test results. Persons may query their test status and results via a unique weblink without requiring an account or log-in. As an alternative to the weblink, a QR (Quick Response) code is provided for easy access.

To secure web communication, creation of new entries, and queuing of test results, our setup consists of different security layers. Only Secure Sockets Layer (SSL)–certified access to the websites is allowed via HTTP Secure (HTTPS). Connection and transferred data are encrypted, and the certificate authority–signed server certificates are used. A reverse-proxy setup forwards the external hostname to a virtual machine within the hospital’s secure network infrastructure. On the virtual server, another reverse proxy is in place to allow for running of the Java application as a nonprivileged user. As the standard HTTP port 80 is privileged, it can only be used by a system user, so running the application as a user with system-wide rights is a security risk. Therefore, proxy settings forward the privileged port to a high port (above 1024) that normal users can control. Hence, no system rights are necessary for managing the application at the operating system level. For the clinical environment setup, we also set up a firewall with specific ban rules—iptables and fail2ban service on a Linux operating system—to prevent brute force attacks on log-in or tracking numbers. Also, network Internet Protocols (IPs) and subnets can be white-listed to allow access to management functions of the application. Thus, other computers and devices are blocked from accessing these functions after failed attempts. For the tracking interface, a brute force attack, such as trying all combinations of possible tracking numbers, is shielded by blocking IPs after too many failed attempts with wrong or nonexistent tracking numbers.

### Query Performance Test

We first performed 1024 simultaneous requests for our performance test using a single machine and a single network connection. Next, we created a mixed data set (ie, interleaved_urls), including 758 available database entries (ie, available_urls) and the same number of unavailable tracking numbers (ie, notavailable_urls). Furthermore, we measured repeated queries of a single available URL, repeated queries of a single unavailable URL, as well as repeats of these two URLs consecutively. That makes six data sets in total, each of which was accessed 1516 times. Queries were carried out once in sequence (ie, ordered) and randomly (ie, random). Also, we tested the two-scenario caching function of the browser (ie, 1filePerRequest) and complete reloading (ie, 23or1filesPerRequest). This means that unchanged files are not reloaded (caching), whereas in the other scenario, all required files and displayed images (eg, flag graphics or Cascading Style Sheets) are reloaded.

For the second scenario, one file is loaded if the tracking number is not available and 23 files are loaded if the tracking number is available. Stress tests were measured using Siege 4.0.4 (JoeDog Software) [37].

### Data Availability

The source code, documentation, and an installation guide are freely available from GitHub [38] under the Eclipse Public License v2.0.

## Results

**Figure 2:**
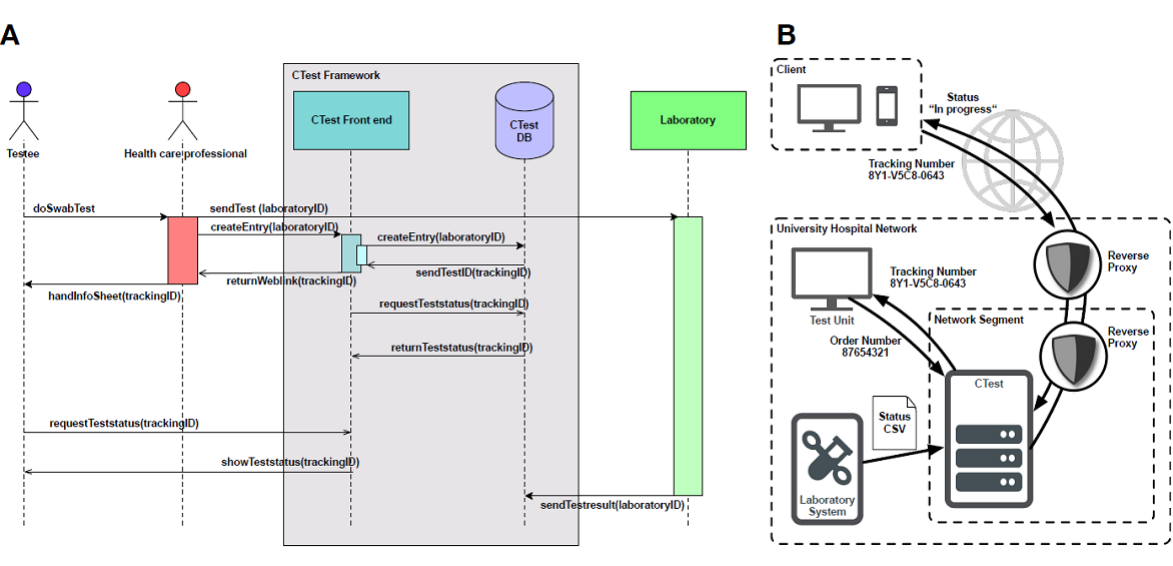
COVID-19 test procedure and setup. (A) The left-hand panel shows the detailed process flow of the COVID-19 tests. CTest (gray) is tightly integrated into the test process. The test framework comprises a web-based front end (turquoise) and a Clojure back end, including a mySQL (Structured Query Language) database (DB) (purple). After the swab test for COVID-19, the health care professional creates a CTest entry via entering the laboratory ID in the CTest front end. Based on that, a secure tracking ID is created by the back end and a corresponding personalized URL is created. CTest then automatically creates a test-specific information sheet for each testee. This sheet contains the test-specific weblink and a QR (Quick Response) code, both based on the tracking ID. The URL does not provide any information to track back the test results to the corresponding testee (blue). Via this URL, a testee can request the corresponding test status at any time. After the analysis in the laboratory, results are transmitted to the CTest database. The CTest front end automatically updates the status on the test-specific weblink based on the corresponding test results. (B) In the right-hand panel, a possible integration scenario of CTest into a clinical infrastructure is shown. CTest runs on a virtual machine within the secured network of a university hospital. The external hostname, with weblinks to the testee's status, is forwarded via reverse proxy. A second reverse proxy forwards the port to the application to a nonprivileged port. Thus, the application does not need to run with a system user. Inside the hospital's network, the test unit communicates to the CTest server to create new test cases via an order number. The CTest server returns back the tracking number and the corresponding weblink and QR code. The laboratory pushes test results as comma-separated values (CSV) files to the CTest server. The test-specific webpage content is then updated according to the test result.

CTest was built based on an existing, proven software stack: TraqBio [30]. It extends TraqBio to the functionality required for COVID-19 tests. We were able to successfully integrate it into the existing clinical testing workflows for SARS-CoV-2 infections in two major German university medical centers—University Hospital Ulm and University Hospital Tübingen—within a few days (Figure 2).

The workflow starts with taking a sample for testing. In the first step, an order number is generated by the testing lab and added as a barcode label to the sample. Analogous to other medical applications, order numbers within the clinical or laboratory information system are unique but do not contain personal information about the patient [39]. This order number is transferred to the CTest system via scanning or typing the number into the dialogue window’s input field. We implemented format restrictions via regular expressions to the input field to minimize incorrect entries. Afterward, CTest generates an unambiguous, nonconsecutive tracking number. Therefore, a cryptographically strong pseudo-random number generator [33] creates 6 bytes that are transferred into a 12-digit character code, including letters from A to F and numbers from 0 to 9. Using this tracking number ensures that no personal information about any testee can be inferred.

**Figure 3:**
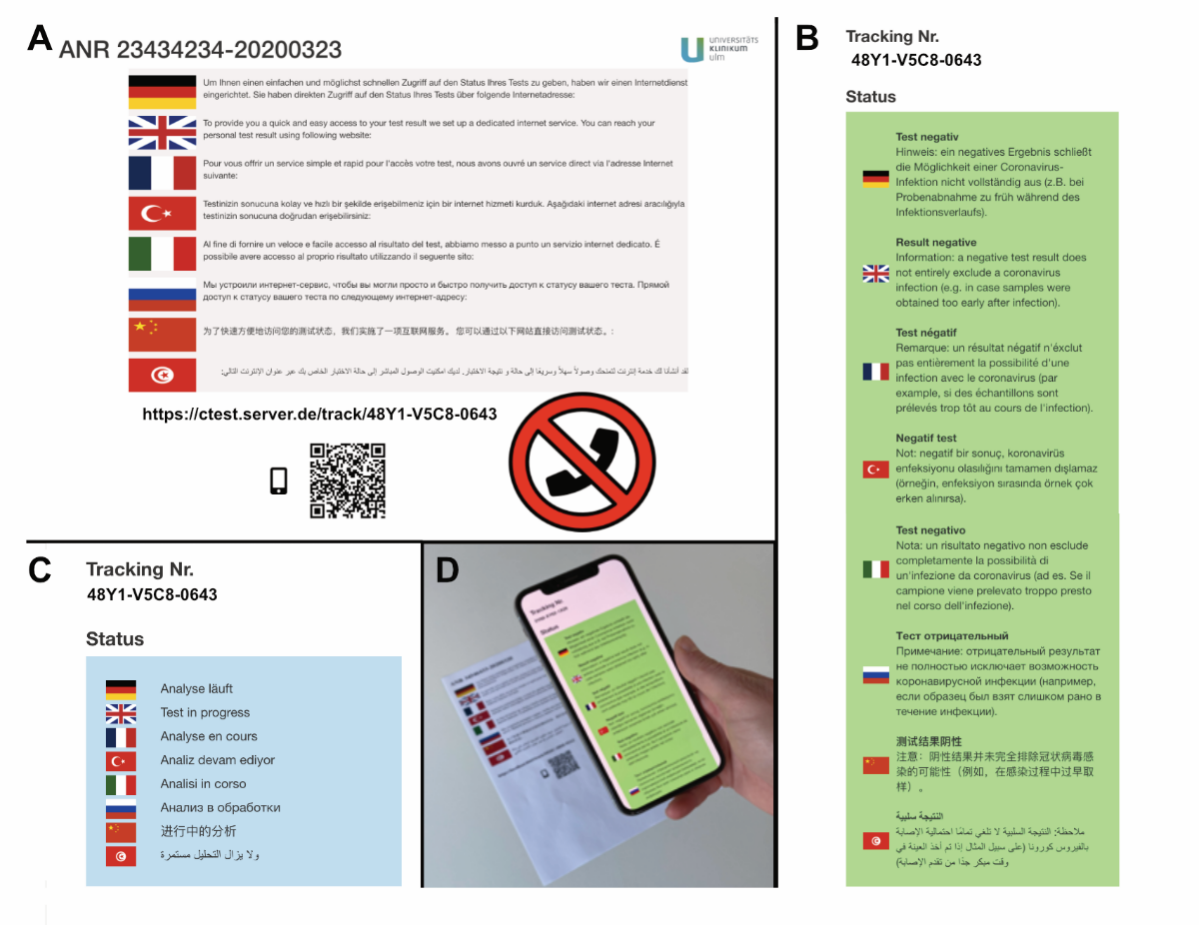
Information for testees. Information concerning access to the test status and its results are given in eight languages. (A) The information sheet handed over to testees informs them of how to access the COVID-19 test results online. (B) The test-specific URL links to a page that includes information about the test result (eg, negative test result) or (C) information stating that the test is still in progress. (D) Scanning the QR (Quick Response) code with a smartphone link takes the testee directly to the current test status or its result.

After taking the sample, the tracking number is given to each testee on a printed sheet, including information on how to access the status of their COVID-19 test (Figure 3, A). After the sample has been processed, the lab system sends updated files as comma-separated values (CSV) files via an encrypted Secure File Transfer Protocol (SFTP) connection to the CTest server. Results in the CSV file are then automatically parsed, backed up, and imported into the CTest database, which leads to an automatic status update of each processed test.

Currently, the CTest system distinguishes multiple potential outcomes: (1) the COVID-19 test is negative (Figure 3, B), (2) the COVID-19 test is still in progress (Figure 3, C), (3) the COVID-19 test is positive, or (4) the COVID-19 test is flawed. As the software is generic, one can implement other outcomes. Phone calls from the health department still inform people who have tested positive for COVID-19. Thereby, officials can inform them about health arrangements and how to avoid further spreading of the virus.

We provide two possibilities to query the status of the COVID-19 test. Testees can either scan a QR code on the information letter they received with a smartphone and get redirected to a test status webpage (Figure 3, D) or they can enter the weblink from the information letter into a web browser directly. The status of the individual test result is automatically displayed in a responsive form on the device (Figure 3, D). We have observed that both possibilities for requesting test results have been used. A total of 30.6% (64,201/209,808) of people preferred to query the test results via a web browser, while the rest of the testees preferred to scan the provided QR code.

To overcome language barriers, we translated information concerning the procedure to obtain test results, and the results themselves, into eight languages: German, English, French, Turkish, Italian, Russian, Chinese, and Arabic. Here, native-language speakers were asked to translate the information and kindly supported the translation process to ensure correct translations. Additionally, in the context of accessibility for medical information and patient empowerment, we paid particular attention to the use of easily understandable language and integration of behavioral recommendations.

**Figure 4:**
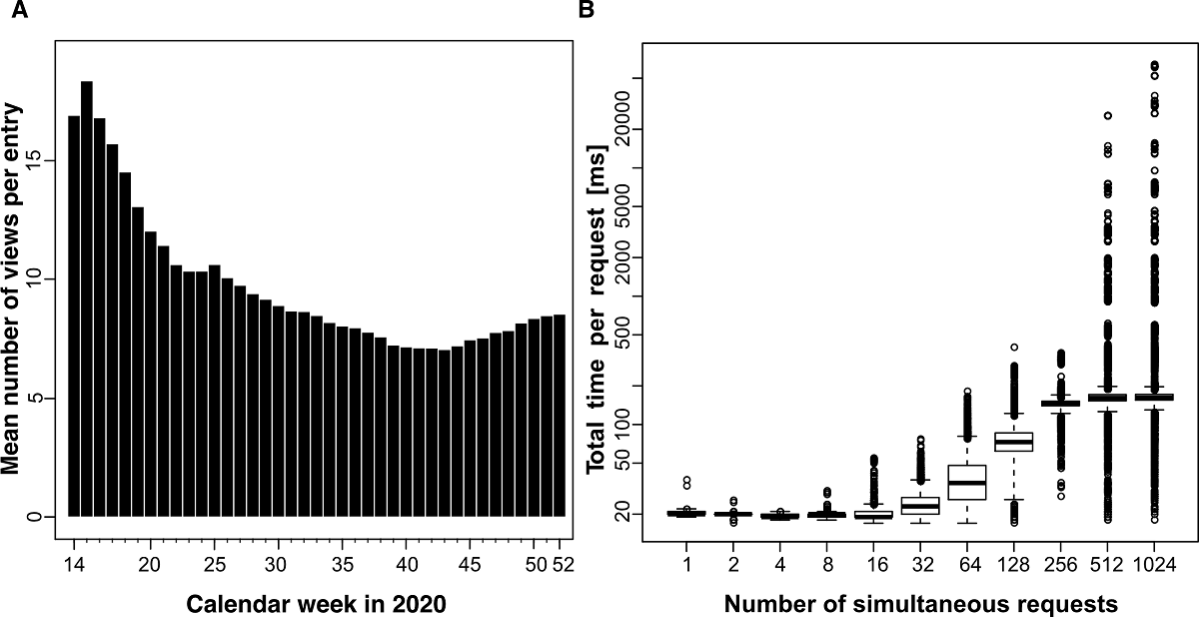
Requests to the CTest server. (A) Since its introduction into the routine at the University Hospital Ulm (Germany) in week 14 (2020), a mean of about 10 (SD 2.8) views per test entry was recorded. The plot shows the mean number of views per entry per calendar week in 2020 as bars. (B) The server can respond within 200 ms to >80% of requests when responding to up to 1024 simultaneous requests. In this simulation, requests were made using a single machine and a single network connection.

Highlighting the reduced burden for clinical staff during the pandemic and the potential scalability of this approach in general, we estimate that there will be a personnel expense according to the derived data. On the one hand, we assume a mean of 10 (SD 2.8) views per entry, as shown in our data (Figure 4, A), and a duration of 30 seconds per access event via CTest. These numbers result in a total time consumption of 5 minutes via the use of CTest. On the other hand, a telephone inquiry is estimated to take 3 minutes if the lines are not busy. Using the number of times that CTest was accessed as the number of phone calls, the estimated amount of time per entry increases to 30 minutes. However, as a more realistic approach, we assume three calls per entry per day, which, at best, would lead to around 9 minutes of inquiry time for the patient.

Furthermore, we checked its performance and robustness in load tests (Figure 4, B, and Figures S1-S6 in Multimedia Appendix 1). Our CTest server can respond within 200 milliseconds to over 80% of requests and within 500 milliseconds to over 90% of requests when responding to up to 1024 simultaneous requests (Figure 4, B). Additionally, we performed stress tests with available and unavailable tracking numbers (Figures S1-S6 in Multimedia Appendix 1). Based on these tests, we are confident that CTest is well-suited to rapid testing demands even if deployed in an ad hoc manner on standard hardware. Its platform independence allows for its deployment on a wide variety of existing infrastructures.

Due to the high number of tests being administered during the peak times of COVID-19 infection waves, CTest was designed to handle large numbers of queries in a short amount of time. At Ulm University Hospital, a maximum of 394 laboratory tests were added to CTest per day. In total, 24,643 tests were added to CTest in 2020. In contrast, we registered up to 5263 access events to the website within one day and 209,808 in total in 2020. Since the introduction of CTest into the clinical routine, there have been around 12 queries performed per test (Figure 4, A). The handling of all these requests via phone calls would lead to communication overload. Consequently, CTest can reduce the burden on clinical staff. On top of that, CTest data at Ulm University Hospital shows that test results are provided to testees within one day.

Another feature of CTest is its functionality for error reporting and statistics. A dedicated *reporter* account is required to access the reporting data. Here, all data are provided in the machine-readable JavaScript Object Notation (JSON) format and the path “/reports/list” can be accessed to get a list of information and error reports. The included information is about the successful backup runs and successful test status imports from the CSV files. Also, the number of test result views per day can be accessed at “/reports/views.” General system information, such as memory consumption and central processing unit usage, is available at “/reports/system.” Furthermore, the sample collection dates are available at “/reports/test-dates” for analysis.

We present CTest as a web interface. In addition to its web interface, CTest facilitates third-party software (ie, apps) access if the testee decides to use it. These apps can query the status using the tracking link with appended “?app=true,” which returns only the test status instead of the complete HTML document.

## Discussion

Fast and efficient communication with patients is the key to effective treatments and avoidance of health-related misunderstandings [40-42]. The COVID-19 crisis has brought a greater level of attention to this topic. In particular, hospital overload and lack of possibilities for direct and in-person communication have highlighted huge limitations for patient empowerment and compliance. On these grounds, we developed a framework to reduce the communication burden and to empower patients. The CTest framework enables an automated system that is always available and that does not require continuous human supervision and office hours. This framework thus reduces the burden on clinical staff involved in the COVID-19 crisis and slows down the spread of the virus by quickly and easily informing people who have been tested with concrete recommendations for action. Furthermore, we wanted to empower testees to obtain their results in a facile and easy-to-access way while, at the same time, ensuring efficient and almost instantaneous and exclusive communication. In the same way, other applications have tried to address patient empowerment and rapid isolation of positive cases [10-14]. Here, home-based self-tests and diagnoses, together with contact tracking, are major pillars that attempt to relieve the burden on public health. These approaches are meant to reduce the number of people with potential COVID-19 infections coming to hospitals for testing and to empower patients by providing insights into their health status [6]. Nevertheless, they still lack reliability and sensitivity [6]. For this reason, it is of relevance to support the information process in the public health context. In general, both aspects together are crucial in the effort to contain the spread of COVID-19. With our approach, we provide a unique workflow to support communication. Even if our implementation attempts to speed up hospital-related processing, we are aware that other steps, from testing to communicating results, are affected by delays. Nevertheless, the presented approach has the potential to support multiple testing locations, therefore, synergistically complementing the effort to provide reliable testing for patients. In this context, our results encourage the application of CTest.

CTest has already been successfully integrated into the hospital information system and captures thousands of COVID-19 tests per week at the University Hospital Ulm and at the University Hospital Tübingen in Germany. As part of the clinical routine, the first analyses of CTest showed a mean of 10 queries per test performed. Even half of that number of telephone inquiries would lead to communication overload. Consequently, the introduction of CTest into the clinical routine could achieve our primary goal of reducing the burden on clinical staff. The open-source license of TraqBio and its clean and simple setup were beneficial for this purpose [30]. This made it possible to implement and integrate the CTest system within a very short period (4 days) into the clinical workflow. Another advantage is that users do not have to create an account to request their test results. Besides, we provide two possibilities for querying the status of the test results. The fact that 30.6% of users queried their test results via QR codes encourages us in this implementation.

Our workload tests demonstrated the ability of CTest to deal with a massive number of parallel access events. These could not be processed via telephone calls. With the numbers estimated in our Results section, one employee could handle around 50 phone calls per 8-hour shift. This fact highlights the potential for increasing the speed of informing testees and for slowing down the virus spread. Using CTest, test results at Ulm University Hospital are available within a mean of one day. In contrast to that, for instance, Omar et al [43] imputed an average delay of 5.56 days in notifying testees with COVID-19–positive results using the conventional way. The estimated results from this study emphasize the reduced amount of time for testees to receive results regardless of business hours and, accordingly, the increasing potential for self-empowerment. Additionally, modeling approaches highlighted the relevance of rapid testing in flattening the infection curve [44,45].

Reducing public health burden is crucial in the final aim of providing digital health support for patient empowerment. In fact, the first consequence for patients of a rapid test response is an overall effect on their stress levels. After being tested, people are worried until they know the result of their test [46]. Notably, in the oncological context, it was shown that stress levels of patients waiting for their diagnosis were comparable to the ones of patients receiving severe diagnostic results [47]. Moreover, not knowing the outcome might tempt testees not to act according to recommendations and, thus, increase the risk of infecting others. Another crucial point we wanted to address with our workflow is ensuring comprehensible and secure communication. For this reason, we implemented translations in eight different languages in CTest to avoid language barriers as much as possible. To ensure the correct understanding of the provided information, we contacted native-language speakers who kindly provided translations. Previous studies outside of the pandemic context already showed that nonnative speakers experienced more stress and uncertainty in communicating with local health care providers. Steinberg and colleagues [42] reported a case study on safe and high-quality health care for children whose parents have limited English proficiencies in the United States. By performing 48 interviews with Latina mothers from two independent cities, they were able to show that these mothers experienced frustration with health care providers. Similar results were obtained from a South African study from Hunter-Adams and Rother [41]. Here, they investigated language barriers between local health care providers and cross-border migrants. Again, interviewed subjects reported frustration in understanding health care indications. Strikingly, some of the participants reported invasive medical procedures performed without explicit consent. Overall, not understanding indications increases fears over unwanted procedures and inaccessibility of health care, which, in the end, affects patient compliance. In the context of the COVID-19 pandemic, these issues would lead to increased communication overload and, consequently, to uncontrolled viral spread. For this reason, we tried to maximize the availability of translation options. The presented workflow also limits the occurrence of third-party translation that would affect patient privacy. In addition, it was shown that partners that try to play the role of interpreter caused additional nonprofessional nonmedical interpretations, again affecting patient empowerment and compliance [41].

In the interest of privacy protection, tracking numbers for test results in our workflow are created based on nonpersonalized order numbers. Based on this implementation, we address big data challenges in personalized medicine [29] and respect the German and European data protection laws. To tackle these data protection issues, clinical or laboratory information systems are often closed-source systems. Therefore, development or integration of new interfaces can be time-consuming. To overcome these barriers, we developed an independent, stand-alone software solution without storing personalized data. Nevertheless, this has the limitation wherein CTest alone cannot automatically transfer positive case results to the public health department. Since this transfer is compulsory in Germany, additional communication still has to be made by employees. In addition, a required step is the input of test results from external sources. Consequently, CTest is independent of existing infrastructures, such as specific laboratory information systems. Data import interfaces can be adapted for a broad range of scenarios (eg, hospitals, independent test centers, or resident physicians).

Currently, CTest is specialized for the query of COVID-19 test results and their status. However, it is a generic framework that can readily be adapted to other queries or to the distribution of different types of test results in all medical fields. Even integrating the CTest system into apps is possible if the HTML view presented here is not desired. For this purpose, the token “?app=true” has to be added after the tracking number. All in all, the idea of CTest is a generalizable approach that can be adapted to various uses in medical communication, such as blood analysis for resident physicians. In this context, an outlook for further development of the software is to have a user interface for configuration. Respective requirements for results, the connected database, and other parameters could be set via a graphic user interface to ease a roll-out into other domains. This might become interesting in the context of electronic prior authorization processes.

## Appendix

Multimedia Appendix 1 Performance and robustness tests.

## Abbreviations

BMBF: Federal Ministry of Education and Research
CSV: comma-separated values
DFG: German Science Foundation
HTTPS: HTTP Secure
IP: Internet Protocol
JSON: JavaScript Object Notation
NFN: Nationales Forschungs Netzwerk
QR: Quick Response
RT-PCR: reverse transcription–polymerase chain reaction
SFTP: Secure File Transfer Protocol
SSL: Secure Sockets Layer
ZIK: Zentrum für Information und Kommunikation
ZIV: Zentrum für Innovative Versorgung
ZPM: Zentren für Personalisierte Medizin
