# Metadata Correction: Patient Empowerment During the COVID-19 Pandemic by Ensuring Safe and Fast Communication of Test Results: Implementation and Performance of a Tracking System

**DOI:** 10.2196/31253

**Published:** 2021-06-21

**Authors:** Gunnar Völkel, Axel Fürstberger, Julian D Schwab, Silke D Werle, Nensi Ikonomi, Thomas Gscheidmeier, Johann M Kraus, Alexander Groß, Martin Holderried, Julien Balig, Franz Jobst, Peter Kuhn, Klaus A Kuhn, Oliver Kohlbacher, Udo X Kaisers, Thomas Seufferlein, Hans A Kestler

**Affiliations:** 1 Institute of Medical Systems Biology Ulm University Ulm Germany; 2 Department of Clinical Chemistry University Hospital Ulm Ulm Germany; 3 Department of Medical Development and Quality Management University Hospital Tübingen Tübingen Germany; 4 University Hospital Ulm Ulm Germany; 5 Comprehensive Cancer Center University Hospital Ulm Ulm Germany; 6 Institute of Medical Informatics, Statistics and Epidemiology Technical University of Munich Ulm Germany; 7 Institute for Translational Bioinformatics University Hospital Tübingen Tübingen Germany; 8 Department of Internal Medicine I University Hospital Ulm Ulm Germany

In “Patient Empowerment During the COVID-19 Pandemic by Ensuring Safe and Fast Communication of Test Results: Implementation and Performance of a Tracking System” (J Med Internet Res 2021;23(6):e27348) the authors noted one error.

In the originally published manuscript, the author Nensi Ikonomi was not credited as an equal contributor. This has been corrected to show that Gunnar Völkel, Axel Fürstberger, Julian D Schwab, Silke D Werle, Nensi Ikonomi, Thomas Seufferlein, and Hans A Kestler all contributed equally to the manuscript.

The correction will appear in the online version of the paper on the JMIR Publications website on June 21, 2021, together with the publication of this correction notice. Because this was made after submission to PubMed, PubMed Central, and other full-text repositories, the corrected article has also been resubmitted to those repositories.

