# Supplementary figures and images for: Patient Empowerment During the COVID-19 Pandemic by Ensuring Safe and Fast Communication of Test Results: Implementation and Performance of a Tracking System
Source: J Med Internet Res. 2021 Jun 7;23(6):e27348. doi: 10.2196/27348 (PMC8189287; doi:10.2196/27348)

# Performance and robustness tests

Figure S1

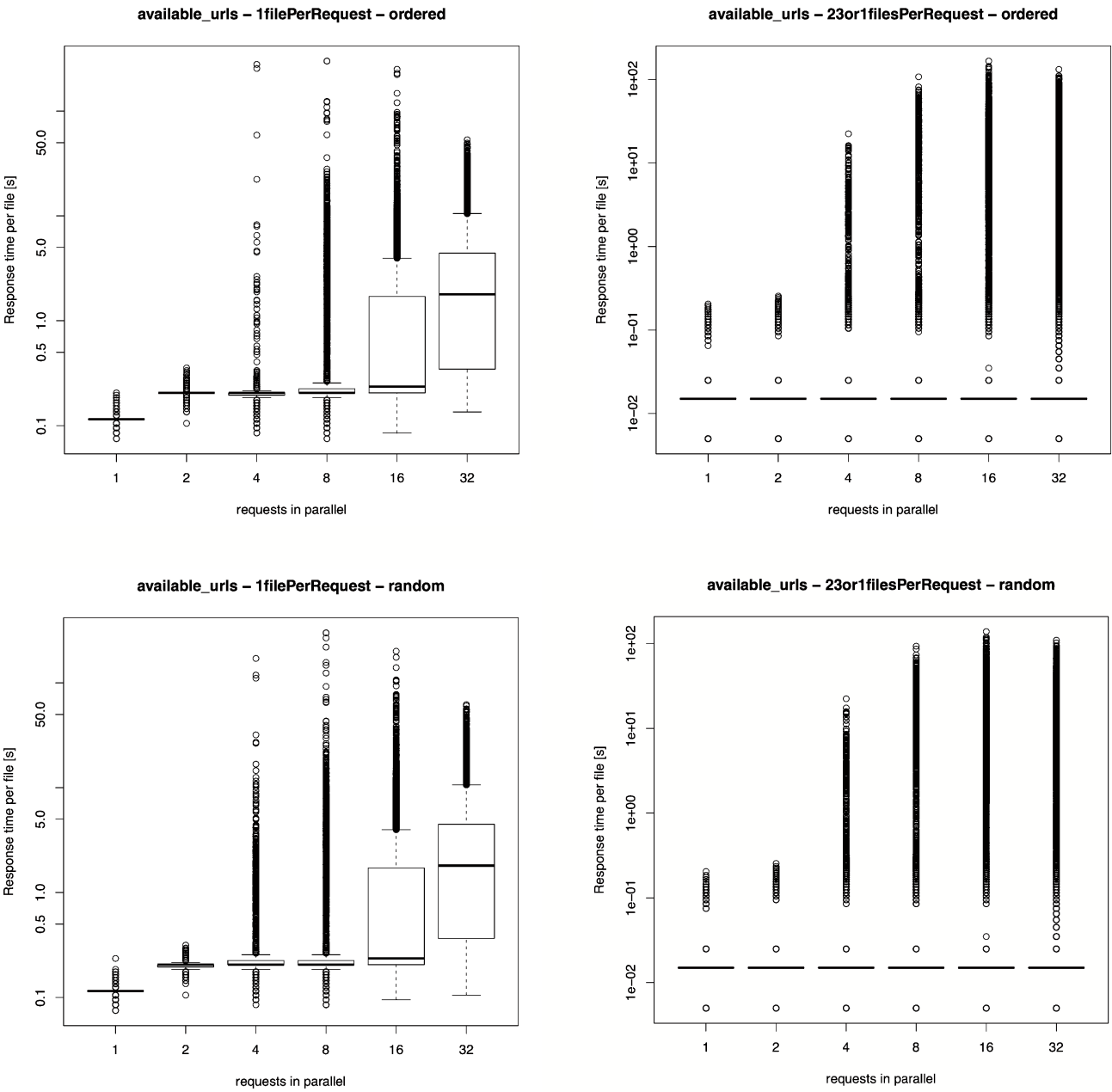

Figure S2

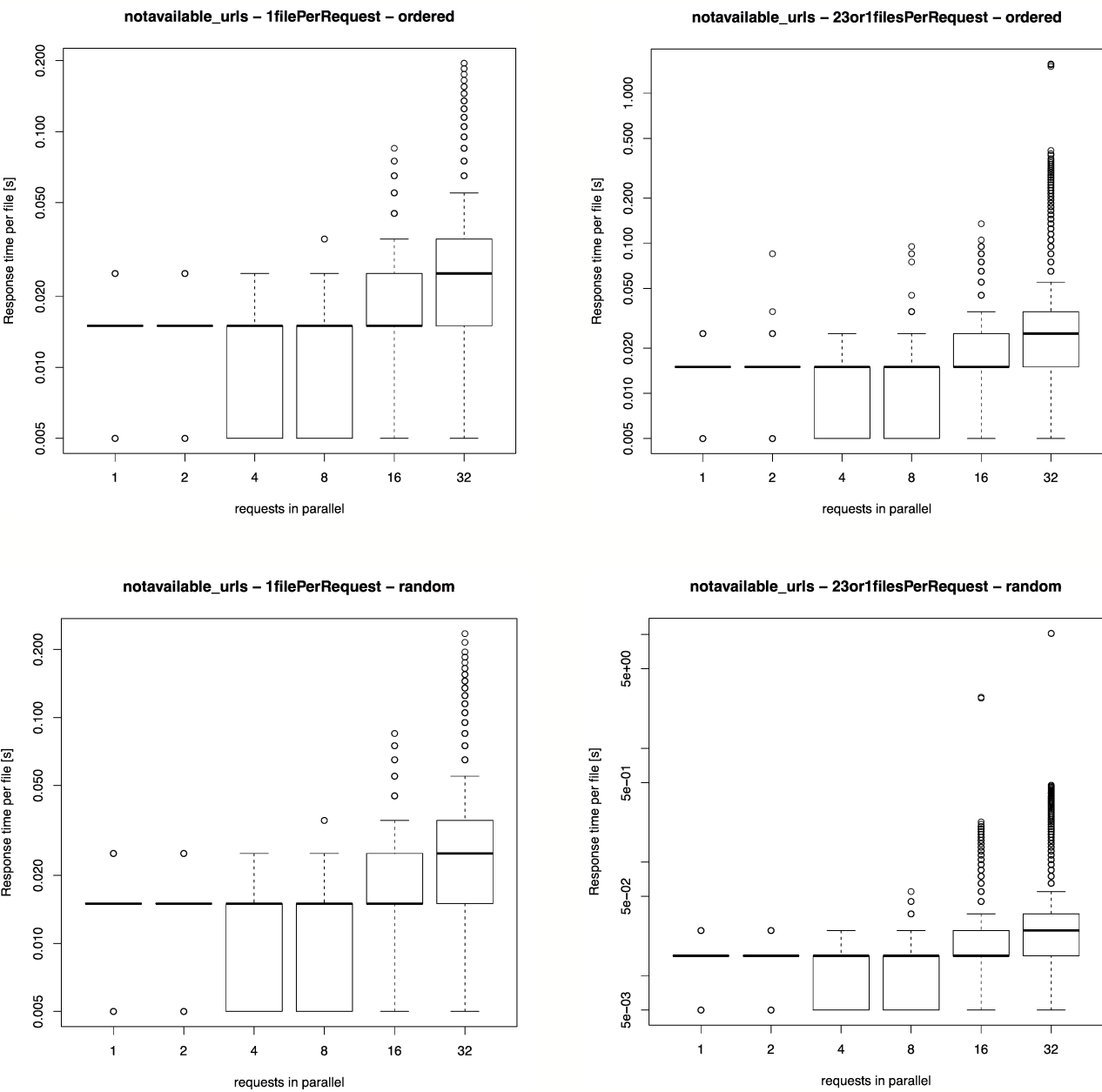

Figure S3

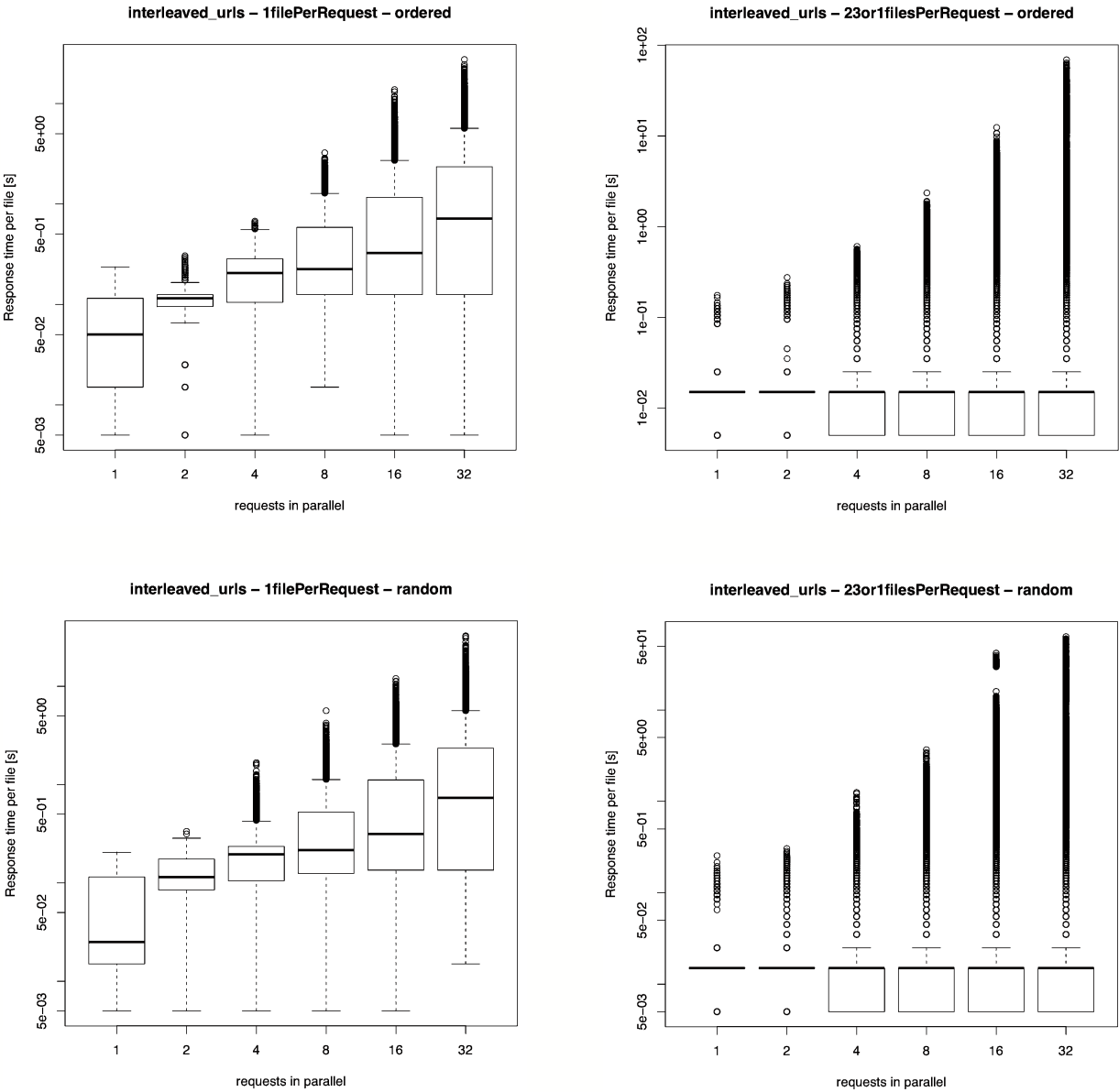

Figure S4

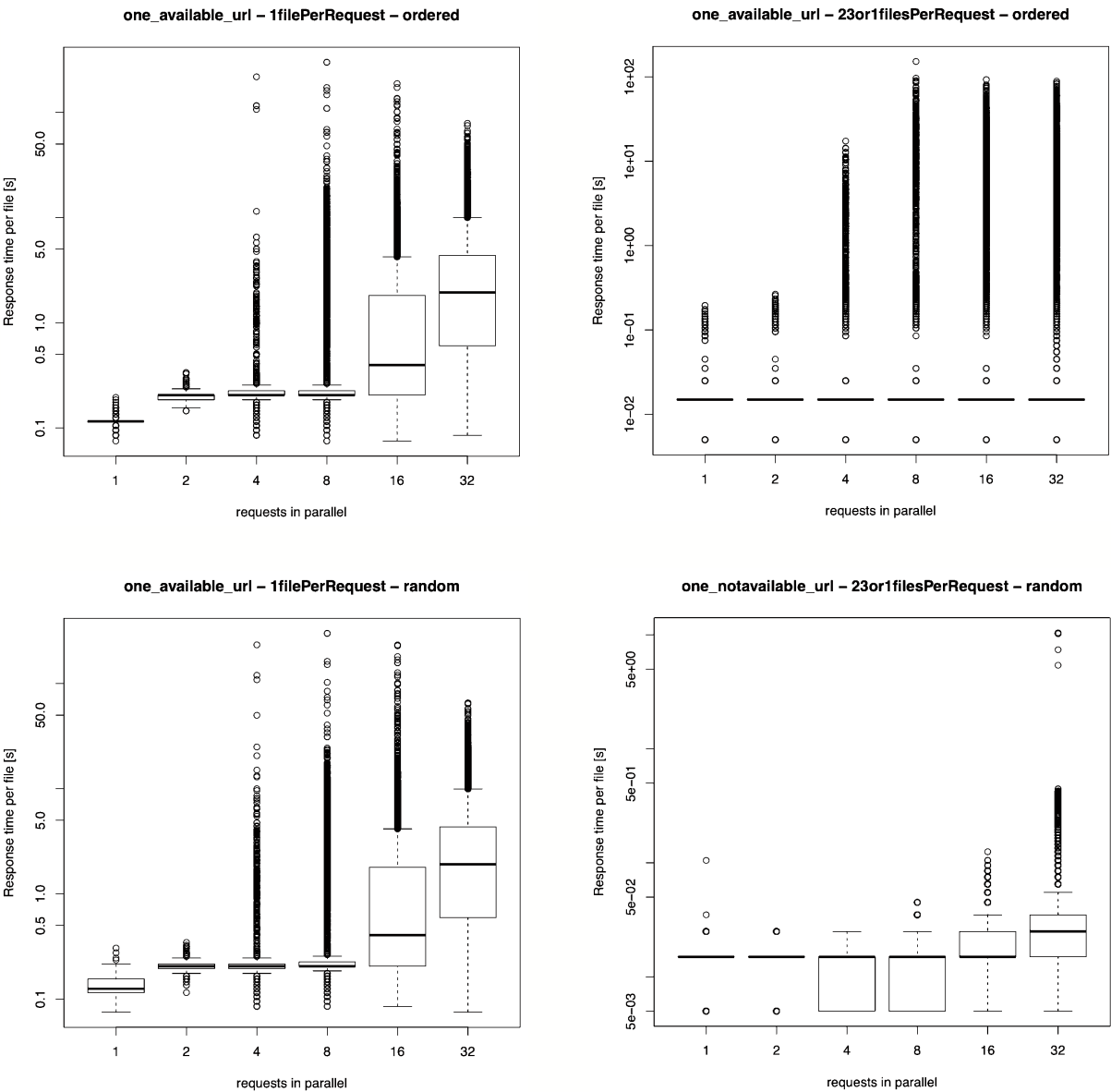

Figure S5

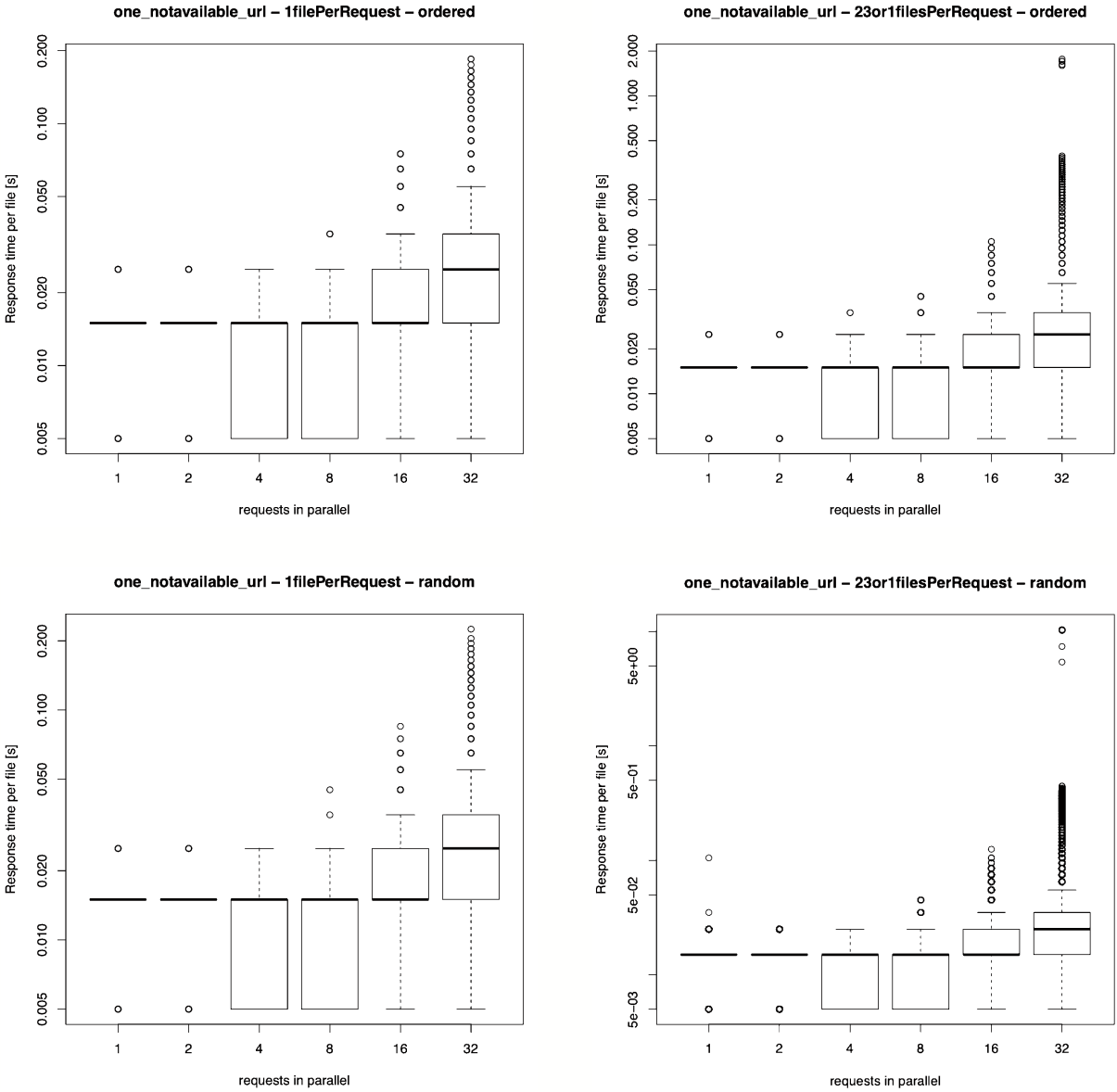

Figure S6

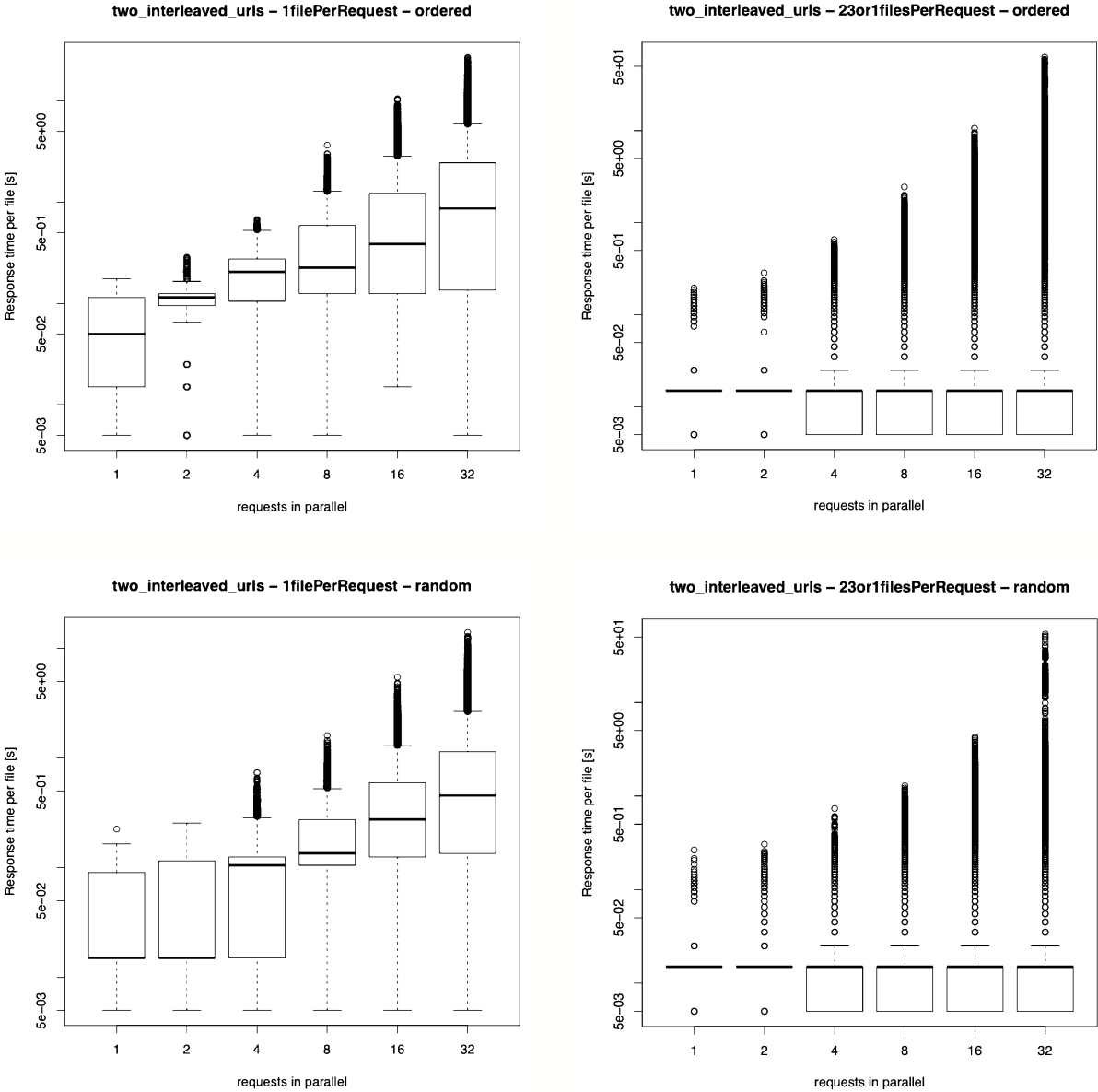

Supplement: Multimedia Appendix 1 [file jmir_v23i6e27348_app1.pdf]
